# Allgemeine Empfehlungen für die Behandlung glomerulärer Erkrankungen – 2023

**DOI:** 10.1007/s00508-023-02265-6

**Published:** 2023-09-20

**Authors:** Martin Windpessl, Philipp Gauckler, Emanuel Zitt, Karl Lhotta, Cihan Ay, Kathrin Eller, Balazs Odler, Irmgard Neumann, Michael Rudnicki, Andreas Kronbichler, Marcus D. Säemann

**Affiliations:** 1 Medizinische Fakultät, JKU, Linz, Österreich; 2 Abteilung für Innere Medizin IV, Klinikum Wels-Grieskirchen, Wels, Österreich; 3 Department Innere Medizin IV (Nephrologie und Hypertensiologie), Medizinische Universität Innsbruck, Innsbruck, Österreich; 4 Abteilung für Innere Medizin III (Nephrologie, Dialyse und Hypertensiologie), Akademisches Lehrkrankenhaus Feldkirch, Feldkirch, Österreich; 5 Klinische Abteilung für Hämatologie und Hämostaseologie, Universitätsklinik für Innere Medizin I, Medizinische Universität Wien, Wien, Österreich; 6 Klinische Abteilung für Nephrologie, Abteilung für Innere Medizin III (Nephrologie, Dialyse und Hypertensiologie), Medizinische Universität Graz, Graz, Österreich; 7 Vasculitis.at, Wien, Österreich; 8 Immunologiezentrum Zürich (IZZ), Zürich, Schweiz; 9 6. Medizinische Abteilung mit Nephrologie & Dialyse, Klinik Ottakring, Wien, Österreich; 10 Medizinische Fakultät, SFU, Wien, Österreich

**Keywords:** Thromboseprophylaxe, Nephrotisches Syndrom, Rituximab, Cyclophosphamid, Pneumocystis jirovecii, Thromboembolism, Nephrotic syndrome, Rituximab, Cyclophophamide, Pneumocystis jirovecii

## Abstract

Glomeruläre Erkrankungen sind mit extrarenalen Komplikationen vergesellschaftet, etwa mit thromboembolischen Ereignissen, einem erhöhten kardiovaskulären Risiko und insbesondere einer deutlich erhöhten Neigung zu Infektionen. Daher ist eine genaue Kenntnis der verschiedenen Immunsuppressiva und ihrer typischen Nebenwirkungsprofile von großer Bedeutung. Während Nephrolog:innen mit Calcineurin-Inhibitoren und Antimetaboliten üblicherweise aus der Transplantationsmedizin viel Erfahrung aufweisen, kommen zwei für die Therapie immunmediierter Nierenerkrankungen relevante Wirkstoffe (Rituximab, in schwerwiegenden Fällen auch Cyclophosphamid) vergleichsweise selten zur Anwendung und werden hier vertiefend besprochen. Außerdem werden praxisgerechte Empfehlungen zur Thromboseprophylaxe beim nephrotischen Syndrom sowie zur Vorbeugung einer wichtigen opportunistischen Infektion, der Pneumocystis jirovecii Pneumonie, gegeben.

## Abstract (en)

Glomerular diseases are associated with extrarenal complications, such as thromboembolism, cardiovascular events and particularly infections. A thorough knowledge of the various immunosuppressants and their associated toxicity profile is therefore of great importance. While nephrologists usually have extensive experience with calcineurin inhibitors and antimetabolites, two other compounds (rituximab, in severe cases cyclophosphamide) are used comparatively infrequently and will be discussed in more detail. Moreover, practical recommendations for the prevention of thromboembolism in states of nephrosis and for the prophylaxis of Pneumcystic jirovecii pneumonia are provided.

## Einleitung

Bei praktisch allen Patient:innen mit glomerulären Nierenerkrankungen sollten begleitend zu einer spezifischen, in der Regel immunsuppressiven Therapie (IST) supportive Maßnahmen etabliert werden. Diese beinhalten in den meisten Fällen eine RAS-Blockade, eine SGLT2-Hemmer-Therapie sowie eine Statintherapie. Dabei sind die allgemeinen Behandlungskautelen (u. a. „*sick-day-rules*“) zu beachten.

Es sollte Normgewicht (Body-Mass-Index [BMI] < 25 kg/m^2^) angestrebt und an Nikotinkarenz appelliert werden. Da häufig eine prolongierte oder wiederholte IST notwendig ist, sollten Zeiten der Krankheitsremission zu einer Grundimmunisierung bzw. zum Schließen von Impflücken genützt werden. Insbesondere werden die sequenzielle Pneumokokkenimpfung (Konjugatvakzine, gefolgt vom Polysaccharidimpfstoff nach > 8 Wochen [bei Personen mit besonders hohem Risiko, dies inkludiert Personen mit Nierenkrankheit]), mit einer Auffrischung nach 6 Jahren sowie eine jährliche Influenza-Impfung empfohlen, außerdem eine Hepatitis B Grundimmunisierung. Es sei auch auf die seit kurzem verfügbare Impfung gegen Herpes Zoster hingewiesen [1]. Bezüglich detaillierter Instruktionen verweisen wir auf die jeweils aktuellen Empfehlungen im Impfplan Österreich [2].

Glukokortikoide (GC) sind Bestandteil vieler Therapieschemata und ihre möglichen metabolischen und infektiösen Nebenwirkungen sollten in der Aufklärung aktiv angesprochen werden. Ein besonderes Augenmerk sollte auf ihre Auswirkungen auf den Muskel- und Knochenstoffwechsel gerichtet werden. Bezüglich spezifischer Empfehlungen zur Prophylaxe (Calcium/Vitamin D-Substitution, ggf. Bisphosphonattherapie) existieren ausführliche Empfehlungen rheumatologischer Fachgesellschaften [3]. Bei einer prolongierten GC-Therapie über der Cushing-Schwellendosis (Prednisolonäqivalent > 7,5 mg/d) ist die Möglichkeit einer Nebenniereninsuffizienz zu beachten.

Vier Themenblöcke werden in der Folge vertiefend dargestellt:

1. Thromboseprophylaxe bei nephrotischem Syndrom
2. Prophylaxe gegen *Pneumocystis jirovecii* Pneumonie
3. Empfehlungen bei Rituximab-Therapie
4. Empfehlungen bei Cyclophosphamid-Therapie

## Thromboseprophylaxe bei nephrotischem Syndrom

Das nephrotische Syndrom geht mit einer Hyperkoagulabilität und daraus resultierend einer erhöhten Neigung zu venösen, aber auch arteriellen Thrombosen einher [4–6]. Beinvenenthrombosen (TVT), Pulmonalembolien (PE) und Nierenvenenthrombosen sind die häufigsten klinischen Folgen, wobei vor allem letztere schwierig zu diagnostizieren sind. Thrombosen können sich in der Akutphase als Niereninfarkt manifestieren, verlaufen aber häufig asymptomatisch und werden oft erst dann erkannt, wenn eine PE aufgetreten ist. In Einzelfällen können sie an ungewöhnlichen Lokalisationen auftreten, beispielsweise als Sinusvenenthrombose oder als Mesenterialvenenthrombose [7, 8]. Die Inzidenz von TVT und PE bei nephrotischem Syndrom wurde früher auf etwa 15 bzw. 10–30 % geschätzt [5], wobei kaum Daten aus aktuellen Kohorten vorliegen und davon ausgegangen werden kann, dass aufgrund wirksamerer Behandlungsmöglichkeiten von Glomerulopathien auch das Auftreten von thromboembolischen Komplikationen rückläufig ist.

Das Thromboserisiko korreliert vor allem mit dem Ausmaß der Hypalbuminämie sowie dem Schweregrad des nephrotischen Syndroms und ist folglich im ersten halben Jahr nach Diagnosestellung am höchsten [5]. Es variiert auch in Abhängigkeit von der für das nephrotische Syndrom verantwortlichen Grundkrankheit. Bestimmte primäre Glomerulopathien (Membranöse Nephropathie [MN], Minimal-Change-Glomerulopathie [MCD], Fokal-segmentale Glomerulosklerose [FSGS]) und einige sekundäre glomeruläre Erkrankungen (Lupus Nephritis Klasse 5, Amyloidose, membranoproliferative GN) zeichnen sich durch eine erhöhte Neigung zu thromboembolischen Komplikationen aus. Bei der MN ist das Risiko am höchsten und auch am besten charakterisiert [5]. Die bei nephrotischen Glomerulopathien (v. a. MCD und FSGS) initial oftmals notwendige hochdosierte Glukokortikoidtherapie stellt einen zusätzlichen Risikofaktor dar [9]. Fast die Hälfte der Thrombosen tritt zum Zeitpunkt der Erstmanifestation auf [10].

Bei der Einschätzung des individuellen Thromboserisikos in Abhängigkeit von der Serumalbumin-Konzentration ist die Methode der Albuminbestimmung im Labor von Bedeutung [11]. Die häufig verwendete Bromkresolgrün-Methode (BKG) überschätzt im Vergleich zur Bromkresolpurpurmethode (BKP) die Albuminkonzentration. In manchen Zentren kommt die Immunturbidimetrie zur Anwendung, diese gilt zwar als Goldstandard, ist aber teurer als die kolorimetrischen Verfahren. Der jeweilige zur Verfügung stehende Test sollte daher bei der Verwendung diverser Risikokalkulatoren und insbesondere beim Vergleich unterschiedlicher Laborwerte beim betroffenen Patienten mitberücksichtigt werden.

Zur Antikoagulation stehen niedermolekulare Heparine (NMH), Vitamin K Antagonisten (VKA) sowie neuerdings direkte orale Antikoagulantien (DOAK) zur Verfügung. Für alle Substanzen ist die Datenlage limitiert. Grundsätzlich ist weiters festzuhalten, dass in der Literatur keine einheitliche Terminologie verwendet wird und auch keine nachvollziehbare Trennlinie zwischen prophylaktischer und therapeutischer Behandlung besteht [12].

### Prophylaktische Antikoagulation

Die Entscheidung für eine *prophylaktische* Antikoagulation sollte auf Basis einer Abschätzung des individuellen Nutzen-Risikoverhältnisses – unter Berücksichtigung relativer und absoluter Kontraindikationen sowie dem Vorhandensein von Risikofaktoren für Thrombosen (siehe Tab. 1) – geprüft werden. Für die Einleitung einer *prophylaktischen* Antikoagulation bei Patienten mit MN und nephrotischem Syndrom kann ein online verfügbares Tool (https://www.med.unc.edu/gntools/) möglicherweise zusätzlich hilfreich sein, wenngleich es nicht validiert ist [13].

**Table 1:** 

Zusätzliche Risikofaktoren	Relative oder absolute Kontraindikationen
Proteinurie > 10 g/d	Präferenz/Adhärenz der Patienten
BMI > 35 kg/m^2^	Blutungsneigung/Blutgerinnungsstörungen
Thrombophilie	–
Schwere Herzinsuffizienz (NYHA III, IV)	–
Rezente größere chirurgische Eingriffe (z. B. orthopädische oder abdominelle Operation)	Hohes Blutungsrisiko, z. B.: – ZNS-Läsion mit Risiko für Hämorrhagie – Gastrointestinale Blutung (Vorgeschichte)
Verlängerte Immobilisation	–

Bei der Entscheidung für eine Antikoagulation kommen auch eine Reihe praxisrelevanter Aspekte zur Überlegung, wobei explizit festgehalten muss, dass keine Evidenz aus kontrollierten Studien vorliegt. Je nach antizipierter Dauer der Antikoagulation und Patient:innenpräferenz kann zwischen NMH (für eine eher kürzeren Zeitraum) oder oralen Antikoagulantien wie VKA gewählt werden. DOAK sollten nur innerhalb des zugelassenen Indikationsbereichs (z. B. Schlaganfallprävention bei nicht-valvulärem Vorhofflimmern, Behandlung von venösen Thromboembolien und Rezidivprophylaxe) und unter Berücksichtigung der Kontraindikationen sowie ihrer pharmakologischen Eigenschaften zum Einsatz kommen.

Weitere praxisrelevante Punkte sind:

- NMH zeichnen sich durch den Vorteil einer nicht relevanten Plasmaeiweißbindung aus, wodurch sich ihr antikoagulatorischer Effekt gut vorhersagen lässt. Limitationen ergeben sich jedoch bei stark eingeschränkter Nierenfunktion (eGFR < 30 ml/min), ausgeprägter Adipositas (BMI > 40 kg/m^2^), starkem Untergewicht und in der Schwangerschaft. In diesen Fällen ist die anti-FXa-Aktivitätsbestimmung empfehlenswert.
- Der HAS-BLED-Score ist für die OAK-Indikation Vorhofflimmern validiert und daher für die Einschätzung des Blutungsrisikos beim nephrotischen Syndrom nur bedingt brauchbar. Obwohl die gleiche Einschränkung auch für den ATRIA-Risikoscore zutrifft, wurde dieser in einem Modell für die MN angewendet [13].
- D‑Dimer-Werte sind im nephrotischen Syndrom erhöht, ohne dass sich klinisch-relevante Thrombosen nachweisen lassen oder sie in der Nachbeobachtung gehäuft aufreten [14].
- Für DOAK beschränkt sich die Datenlage auf unkontrollierte Einzelfallberichte und Fall-Serien. Die Erfahrung mit diesen Substanzen ist aufgrund der kürzeren Verfügbarkeit und geringeren Patientenzahl bislang noch begrenzt [12]. Aus pharmakologischen Überlegungen könnten sich jedoch Vorteile gegenüber Marcoumar ergeben. Coumadin oder Marcoumar sind zu 99 % an Albumin gebunden, sodass bei ausgeprägter Hypalbuminämie bei nephrotischem Syndrom eine deutlich höhere Plasmaclearance gegeben ist, mit bis zu 2 × geringerer Halbwertszeit [15]. Häufige INR-Kontrollen werden daher empfohlen, insbesondere bei steigenden Serumalbumin-Werten (als Frühzeichen der einsetzenden Remission des nephrotischen Syndroms), um in dieser Phase dann Überantikoagulation und steigendes Blutungsrisiko zu verringern. DOAK variieren in ihrer Eiweißbindung (Dabigatran 35 %, Edoxaban 55 %, Rivaroxaban 92–95 %, Apixaban 87 %) und renalen Ausscheidung (Dabigatran 80 %, Rivaroxaban 33 %, Edoxaban 50 %, Apixaban 27 %) und damit Dosierungsabhängigkeit von der Nierenfunktion [16].

Auf Basis der vorhandenen, wenngleich limitierten Evidenz, klinischen Erfahrung, pathophysiologischen Überlegungen und Praktikabilitätsgründen empfehlen wir folgendes Vorgehen:

Bei Serum-Albumin < 2,5 g/dl (BKG; bei Verwendung von BKP oder Immuno-Assay < 2 g/dl) und hohem Thromboserisiko (entweder MN als primäre Grunderkrankung des NS oder zusätzlicher Faktoren für Thrombosen) soll eine Thrombose*prophylaxe* in Erwägung gezogen werden, wenn das Blutungsrisiko nicht erhöht ist.

Bei Serum-Albumin < 2,0 g/dl sollte unabhängig von der zugrundeliegenden Nierenerkrankung eine Thrombose*prophylaxe* aufgrund des nephrotischen Syndroms in Erwägung gezogen werden, wenn das Blutungsrisiko nicht erhöht ist.

Möglichkeiten der Thromboseprophylaxe:

- Niedermolekulares Heparin (NMH; z. B. Enoxaparin 4000 UE (40 mg) sc 1 × täglich; bei eGFR < 30 ml/min 2000 IE (20 mg) sc 1 × täglich) [17, 18].
  Ziel-aFXa-Aktivität (gemessen 4 h nach Gabe nach mind. 3–4 Einzelgaben) 0,3–0,5 IU/ml (nicht > 0,8 IU/ml!).
- VKA (Phenprocoumon, Acenocoumarol) mit einer Ziel-INR von 1,5–2,5.
  Diese Option ist insbesondere bei voraussichtlich länger persistierender Hypalbuminämie, d. h. > mehrere Wochen oder bei Patientenwunsch (Ablehnung der täglichen Subkutaninjektion) zu überlegen. Vorteile einer Therapie mit VKA sind die langjährige Erfahrung mit dieser Substanz und die etablierte Möglichkeit einer Messung der Intensität der Antikoagulation. Andererseits ist die Zeit im therapeutischen Bereich (*time in therapeutic range*) selbst in gut kontrollierten Kohorten (z. B. Patienten mit Vorhofflimmern) suboptimal. Bei zwangsläufig schwankenden Serum-Albumin-Werten ist eine ausreichend stabile Antikoagulation erschwert, weshalb engmaschige INR-Kontrolle notwendig sind.
- Der Einsatz von DOAK zur Thromboseprophylaxe ist „off-label“. Es liegen keine Studien vor, die die verschiedenen grundsätzlichen Strategien zur Antikoagulation miteinander verglichen oder randomisiert gegen Placebo getestet haben. Daher kann derzeit keine dieser Strategien in der Thromboseprophylaxe bei Patienten mit nephrotischen Syndrom und ausgeprägter Hypalbuminämie bevorzugt werden.
- Im Serum-Albumin-Bereich zwischen 2,5–2,9 g/dl liegen zumindest für die MN auch Beobachtungsdaten für Aspirin (ASS) in einer Dosierung von 75 mg/d vor. ASS kommt vor allem bei intermediärem Blutungsrisiko als Therapieoption in Frage [17, 19].

Die Dauer der prophylaktischen Antikoagulation sollte sich im Wesentlichen an der Dauer des nephrotischen Syndroms orientieren, wobei eine regelmäßige Nutzen-Risiko-Abwägung erfolgen sollte. Steigt das Serum-Albumin wieder anhaltend über 2,5–3,0 g/dl an, kann die Antikoagulation beendet werden, vorausgesetzt, es liegen keine anderen Gründe für eine gerinnungsaktive Therapie vor.

## Prophylaxe gegen *Pneumocystis jirovecii* Pneumonie

Eine *Pneumocystis jirovecii* Pneumonie (PJP) ist eine potenziell lebensbedrohliche opportunistische Infektion und wird durch einen atypischen, ubiquitären Pilz der Gattung *Pneumocystis* hervorgerufen. Das Risiko dieser Komplikation korreliert mit dem Grad der Immunkompetenz, beispielsweise haben Patienten mit einer fortgeschrittenen HIV-Infektion ein deutlich erhöhtes Risiko. Dies spiegelt sich auch in der Autoimmunität wider, wobei Erkrankungen mit systemischem Charakter und v. a. schwerer Beeinträchtigung des Allgemeinzustands (z. B. ANCA-assoziierte Vaskulitis [AAV], systemischer Lupus erythematodes [SLE], anti-GBM Erkrankung) ein hohes Risiko aufweisen.

Die beste Evidenz für eine PJP-Prophylaxe gibt es für die AAV. Hier wird in den EULAR 2022 Empfehlungen eine PJP-Prophylaxe für alle Patienten, welche Rituximab, Cyclophosphamid, und/oder eine höhere Steroiddosis erhalten, empfohlen [20]. Eine höhere Dosis an Steroiden wird definiert als eine wochenlange Verschreibung von ≥ 30 mg Prednisolonäquivalent pro Tag. Diese Empfehlung basiert auf einer großen Kohortenstudie aus Korea [21]. Mehrere Observationsstudien haben allerdings zuletzt von einer effektiven Reduktion schwerer Infekte durch eine prophylaktische Dosis von Trimethoprim-Sulfamethoxazol (T/S) berichtet [22–24]. Eine Sub-Analyse der RAVE-Studie (Rituximab versus Cyclophosphamid/Azathioprin) untersuchte spezifische Risikofaktoren schwerer Infekte [25]. Eine Therapie mit T/S war anderen prophylaktischen Maßnahmen zur Reduktion von PJP signifikant überlegen; daher sollte bei guter Verträglichkeit T/S priorisiert werden.

Für andere in diesem Band diskutierten Entitäten gibt es weniger Evidenz. In der TESTING Studie (Hochdosis Methylprednisolon) gab es 3 Fälle einer PJP, wobei Berichte einer PJP als Komplikation von IgAN außerhalb von China äußerst selten sind. PJP scheint in manchen Zentren weit verbreitet zu sein und bei generellen Ausbrüchen sollte eine Prophylaxe auch in einem Setting mit einem niedrigen Risiko erwogen werden [26]. Eine rezente Untersuchung von 1168 Patienten mit Riesenzellarteritis hat während einer Nachbeobachtung von 547 Patientenjahren keinen Fall von PJP berichtet [27]. Ähnlich selten scheint eine PJP bei Patienten mit MCD, FSGS, MN und IgAN in Mitteleuropa zu sein, und deshalb erscheint uns eine Prophylaxe bei diesen Patientengruppen als nicht indiziert. In derartigen Szenarien dürften die potenziell schwerwiegenden Nebenwirkungen von T/S einen möglichen Nutzen überwiegen, nachdem Komplikationen wie Stevens-Johnson Syndrom, isolierte Leukopenien, schwere Panzytopenien sowie Leberfunktionsbeeinträchtigungen berichtet wurden [28, 29]. Für andere Entitäten wie Lupus Nephritis oder MPGN gibt es weniger klare Empfehlungen als für die AAV. Bei der Lupus Nephritis würden wir für die Dauer der Induktionsphase, eine Prophylaxe empfehlen, v. a. wenn die Steroiddosis ≥ 20 mg pro Tag beträgt [30]. Die Datenlage für diese Empfehlung ist allerdings limitiert. Zudem gibt es keine Informationen zur Effektivität von T/S zur Prophylaxe schwerer Infekte bei diesen Entitäten.

T/S ist in Österreich als Bactrim®, Lidaprim® und Eusaprim® verfügbar. Die „forte“ Formulierung bezieht sich auf die Trimethoprim-Komponente und entspricht 160 mg (→ 160/800 mg T/S).

Mögliche Dosierungen (Prophylaxe): 160/800 mg 3 × pro Woche; bei eGFR < 30 ml/min/m^2^: halbe Dosis. Empfehlungen zur PJP-Prophylaxe sind in Tab. 2 zusammengefasst.

**Table 2:** 

Entität(en)	Empfehlung
ANCA-Glomerulonephritis	T/S sollte in der Induktionsphase gegeben werden; weniger Evidenz während der Erhaltungsphase T/S reduziert die Frequenz von schweren Infekten (im Gegensatz zu anderen prophylaktischen Maßnahmen, z. B. Dapson, Atovaquon) Unklar: optimale Dosis (480 mg oder 960 mg 3‑mal wöchentlich)
Lupus Nephritis	T/S kann während der Induktionsphase gegeben werden, vor allem bei multimorbiden Patienten Unklar: Evidenz, Nebenwirkungsprofil beachten
MPGN	T/S kann während der Gabe von Immunsuppressiva (wie z. B. Rituximab) oder länger-andauernde Episoden einer Hochdosis Steroidtherapie gegeben werden Unklar: Evidenz, Nebenwirkungsprofil beachten
Nephrotische Erkrankungen	Keine Empfehlung
IgA Nephropathie	Keine Empfehlung

## Rituximab (RTX) und andere anti-CD20-Antikörper

RTX ist ein intravenös verabreichter monoklonaler, chimärer Antikörper gegen das CD20-Oberflächenantigen auf B‑Lymphozyten (anti-CD20-Antikörper [anti-CD20-Ak]); der Wirkstoff führt zu einer bis zu 12 Monaten anhaltenden B‑Zell-Depletion und wird außerhalb der Zulassung (AAV) für verschiedene immun-mediierte Nierenerkrankungen sowie im Rahmen von AB0-inkompatiblen Nierentransplantationen verwendet [31]. Neuere, sogenannte „*next generation*“ anti-CD20-Ak (z. B. Obinutuzumab) kommen bei refraktären Verläufen bereits jetzt zum Einsatz und dürften in den nächsten Jahren noch an Bedeutung gewinnen.

Obschon eine anti-CD20-Therapie ein grundsätzlich günstiges Nutzen/Risikoprofil aufweist, ist sie mit einem erhöhten Infektionsrisiko vergesellschaftet, weshalb vor und während der Behandlung einige Punkte zu beachten sind. Eine weitere häufige Nebenwirkung ist die sogenannte Infusionsreaktion (IR), welche am häufigsten bei Erstgabe auftritt [31].

Eine IR ist durch ein Zytokin-Freisetzungs-Syndrom bedingt (typische Manifestationsformen sind grippale Symptome wie Myalgien und Fieber). Im nephrologischen Kontext treten sie mit einer Häufigkeit von etwa 25 % auf und verlaufen in der Regel mild, sodass die Infusion gegebenenfalls nur pausiert und konsekutiv mit der halben Geschwindigkeit fortgesetzt werden kann. Da Blutdruckabfälle häufig vorkommen, kann im Vorfeld das Pausieren einer vorbestehenden Hochdrucktherapie erwogen werden [31].

Eine genuine allergische Reaktion mit urtikariellem Exanthem, Dyspnoe und/oder Bronchospasmus ist zwar deutlich seltener, verbietet jedoch eine Re-Exposition. In derartigen Fällen ist den üblichen Anaphylaxie-Richtlinien zu folgen.

Folgende Punkte sind im Kontext einer anti-CD20-Therapie besonders zu beachten:

- Eine aktive Hepatitis B Erkrankung stellt eine Kontraindikation dar, aber auch nach durchgemachter Hepatitis B-Infektion besteht ein hohes Risiko für eine Reaktivierung, sodass einerseits eine entsprechende serologische Diagnostik vorab zwingend, andererseits bei „Z. n. Hepatitis B“ eine Prophylaxe obligat ist. Diese sollte idealerweise eine Woche vor Behandlungsbeginn etabliert werden (z. B. Tenofovir oder Entecavir). Aufgrund der langen Wirkdauer von RTX sollte die Prophylaxe zumindest für ein Jahr nach letzter RTX-Gabe erfolgen. Eine Absprache mit Hepatolog:innen für Therapieplanung ist sinnvoll [1, 31].
- RTX ist mit einem sekundären Immunglobulinmangel (Hypogammaglobulinämie) assoziiert, sodass vor Therapiestart ein Ausgangsbefund bestimmt werden sollte. Weisen Patienten bereits zu Beginn niedrig-normale Werte auf, besteht ein höheres Risiko für schwerere Hypogammaglobulinämie. Eine Graduierung erfolgt nach mild (IgG > 5 g/l bis < 7 g/l), moderat (IgG > 3 g/l bis < 5 g/l) und schwer (≤ 3 g/l). Eine intravenöse Immunglobulingabe (IVIG) ist nur bei rezidivierenden schweren infektiösen Komplikationen angezeigt. Wir monitieren Immunglobuline bei laufender RTX-Therapie quartalsmäßig [31].
- Ein erhöhtes Risiko für eine opportunistische Infektion mit *Pneumocystis jirovecii *besteht vor allem bei Multisystemerkrankungen (AAV), insbesondere, wenn eine pulmonale Involvierung vorliegt. Bei renal-limitierten Entitäten und isolierter RTX-Therapie (beispielsweise MN, MCD oder FSGS) erachten wir eine Prophylaxe mit TMP/SMX nicht obligat [1].
  Bezüglich Details zur *Pneumocystis*-Prophylaxe verweisen wir auf den entsprechenden Abschnitt.
- Aufgrund eines prolongiert schlechten Impfansprechens nach anti-CD20-Therapie sollte im Vorfeld der Impftstatus erhoben werden und allfällige Impflücken nach Möglichkeit geschlossen werden, insbesondere gegen Covid 19, Pneumokokken, Hepatitis B sowie – in Abhängigkeit der Jahreszeit – Influenza [1].

Eine entsprechende Patientenaufklärung ist obligat und kann von der ÖGN-Homepage für die „nephrologischen Indikationen“ heruntergeladen werden (Microsoft Word – Patientenmerkblatt_RTX.docx (webflow.com)).

Die Verabreichung ist grundsätzlich ambulant oder tagesklinisch möglich. Bei Erstgabe ist auch aufgrund der langen Dauer (4 bis 5 h) und der Logistik im Vorfeld zumeist eine stationäre Aufnahme sinnvoll. Bei guter Verträglichkeit können die weiteren Gaben bei entsprechender Infrastruktur auch ambulant erfolgen, zumal die Infusionsgeschwindigkeit konsekutiv erhöht werden kann.

Es gibt keine einheitliche Dosierung für „nephrologische Indikationen“ und beide etablierten Schemata, „hämatologisch“ = 4 Gaben zu je 375 mg/m^2^ oder „rheumatologisch“ = 2 Gaben zu je 1 g absolut werden verwendet. Für Erhaltungstherapien (beispielsweise AAV) kommen auch Dosierungen mit 500 mg zur Anwendung.

Eine zwingende Indikation zum Nachweis einer B‑Zell-Depletion mittels Lymphozytentypisierung besteht nicht, kann in Einzelfällen jedoch nützlich sein, insbesondere bei nephrotischen Nierenerkrankungen, bei denen ein hoher renaler Verlust des Antikörpers eine eingeschränkte Wirksamkeit nach sich ziehen kann. Auch in der Behandlung der Lupus Nephritis ist eine ausbleibende B‑Zell-Depletion mit einem schlechten Therapieansprechen vergesellschaftet.

Prinzipiell empfiehlt sich bei RTX-Gabe ein strukturiertes Vorgehen mittels SOP, diese kann wie folgt aussehen:

### SOP Rituximab

- Gegenanzeigen laut Gebrauchsinformation: Aktive Infektionskrankheit, Herzinsuffizienz NYHA IV, Schwangerschaft, bekannte Allergie gegen den Wirkstoff
- Allerdings kann Rituximab bei schweren Verläufen nach sorgfältiger Nutzen-Risiko-Abwägung sowohl bei Infektionskrankheiten als auch in der Schwangerschaft verabreicht werden (relative Kontraindikationen) [32–34].
- Kautelen: Notfallwagen in Reichweite, Monitierung durch Pflegepersonal laut eigener SOP

#### Vor Rituximab-Gabe

- Ärztliches Aufnahmegespräch, Vitalparameter messen und dokumentieren
- Venflon
- Laborbefunde: Aufnahmelabor, Immunglobuline (IgG/IgA/IgM – falls keine Vorbefunde < 3 Monate vorliegend), *Harnbefund*
- **Obligat: Hepatitis-Serologie** (inklusive HBsAk) – bei positiven HBsAg oder HBsAk Rücksprache mit Hepatologen bezüglich virostatischer Therapie (nach Möglichkeit 1 Woche vor Rituximab-Gabe beginnen)
- Zusätzliche (Index)Serologie: SARS-CoV 2, HIV, CMV, EBV, sollten als Ausgangsbefunde vorliegen
- Patient und Arzt unterschreiben Dokument „Patientenmerkblatt: Rituximab bei Nierenerkrankungen“ (Original für Krankengeschichte, Patient bekommt eine Kopie ausgehändigt)
- Ärztliche Freigabe zur Rituximab-Infusion nach Ausschluss von KI und Prüfung der Laborbefunde
- Prämedikation 30 min vor Rituximab-Infusion (falls nicht anders angeordnet)
  - 1000 mg Paracetamol per os
  - (Methyl)Prednisolon 50–100 mg
  - Diphenhydramin 30 mg Kurzinfusion über 15 min (Trägerlösung: 0,9 % NaCl 100 ml)

Venflon spülenVerabreichung nach Infusionsschema und mit regelmäßigen Kontrollen durch Pflegepersonal laut gesonderter SOP.

## Cyclophosphamid

Cyclophosphamid (CYC), ein Zytostatikum mit alkylierender Wirkung, führt zur Hemmung der DNA-Replikation und folglich zur Apoptose. Diese Effekte zeigen sich vor allem auf die zellvermittelte Immunantwort und spiegeln sich im Laborbefund in einer deutlichen Abnahme der Lymphozyten wider. Zusätzlich zur Beeinflussung der zellulären Immunität beeinflusst CYC auch die Aktivierung und Differenzierung von B Zellen und daher auch Antikörper-vermittelte Prozesse [35].

Aufgrund der zunehmenden Verfügbarkeit alternativer Immunsuppressiva hat CYC in der Nephrologie mittlerweile an Stellenwert eingebüßt. Nichtsdestotrotz bleibt es in einigen Indikationen eine Therapieoption. Die Applikation von CYC erfolgt entweder in Tablettenform (Endoxan® Dragees à 50 mg) oder intravenös.

In nephrologischer Indikation gibt es drei wesentliche Schemata für die parenterale Anwendung (siehe Tab. 1). Bei der Lupus Nephritis (LN) hat sich das „Euro Lupus Schema“ in Mitteleuropa etabliert [36]. Das „NIH-Schema“ mit deutlich höherer kumulativer CYC-Dosis wird bei seltenen lebensbedrohlichen Manifestationen, wie etwa ZNS- oder Herzbeteiligung, eingesetzt [37]. Bei der ANCA-assoziierten Vaskulitis (AAV) spielt eine intravenöse Gabe von CYC nach wie vor eine bedeutende Rolle.

Analog zur RITUXVAS Studie, die Rituximab (4-mal 375 mg/m^2^) mit CYC (2 intravenöse Applikationen) gegen eine Standardtherapie untersucht hat, ist ein „2 × 2“ (2 × 1 g Rituximab, 2 × CYC) zur Induktionstherapie beliebt, da dadurch die kumulative Steroidexposition reduziert werden kann [38, 39]. Eine orale CYC-Therapie (2 mg/kg Körpergewicht, maximal: 200 mg pro Tag) ist bei der AAV nur mehr von untergeordneter Bedeutung, wird jedoch in vielen Ländern noch in der Therapie der membranösen Nephropathie eingesetzt.

Nach dem „modifizierten Ponticelli-Schema“ sollte in den Monaten 2, 4 und 6 eine orale CYC-Therapie (2 mg/kg Körpergewicht, maximale Dosis 175 mg) erfolgen. Diese Therapie wird in Österreich nur in Einzelfällen verwendet. In der Behandlung der anti-GBM-Erkrankung ist die orale Gabe hingegen Therapiestandard.

Die CYC-Dosis muss an Nierenfunktion, Alter und in weiterer Folge Leukozytennadir, d. h. Abfall der Leukozyten (siehe Tab. 3), angepasst werden. Die Leukozyten sollten zwischen Tag 10 und 14 nach CYC-Gabe gemessen werden [40]. Dieses Protokoll der CYCLOPS-Studie diente auch als Vorlage für die deutsche S1-Leitlinie zur Therapie der AAV [40, 41].

**Table 3:** 

Schema	Euro Lupus (LN)	NIH (LN)	CYCLOPS (AAV)
Cyclophosphamid-Dosis pro Gabe	500 mg	0,5–1 g/m^2^	15 mg/kg Körpergewicht (2,5 mg/kg Dosisreduktion bei einem Alter > 60 Jahre; 5 mg/kg Dosisreduktion bei einem Alter > 70 Jahre, weitere Dosisreduktion abhängig von Nierenfunktion (Kreatinin 3,4–5,7 mg/dl: weitere 2,5 mg/kg Dosisreduktion)) Reduktion um 20 % (Leukozytennadir 2–3 × 10^9^/l); Reduktion um 40 % (Nadir 1–2 × 10^9^/l)
Gaben	6‑mal	6‑mal (ursprünglich im Anschluss alle 3 Monate)	6–10 Gaben (abhängig vom Krankheitsstatus); heutzutage auch flexibel (z. B. 2 Gaben, wenn gleichzeitige Applikation von Rituximab)
Abstand zwischen den Gaben	2 Wochen	4 Wochen	Die ersten 3 Gaben im Abstand von 2 Wochen, danach alle 3 Wochen

Neben der Hämatotoxizität (siehe oben) gibt es noch weitere potenzielle Komplikationen der CYC-Therapie [35].

1. **Fertilität:** Mit zunehmender CYC-Exposition (kumulative Dosis) nimmt die Fertilität bei Frauen und Männern ab, daher sollte ein kurzfristiger Einsatz mit geringen Dosen in Erwägung gezogen werden. Eine Beratung mit der Gynäkologie ist vorab in solchen Situationen empfehlenswert, um das weitere Vorgehen (Kryokonservierung von Spermien, Therapie mit GnRH oder LH-RH Analoga) zu besprechen.
2. **Hämorrhagische Zystitis:** Die Harnblasentoxizität wird durch den CYC-Metaboliten Acrolein verursacht. Eine hämorrhagische Zystitis kann durch eine sehr hohe Dosis von CYC bzw. durch eine hohe kumulative Dosis ausgelöst werden. Das Risiko eines Malignoms der Harnblase war früher eine gefürchtete Komplikation, wobei dies durch die deutlich geringeren Gesamtdosen moderner Therapieprotokolle nicht mehr gehäuft auftritt. Der Einsatz von Uromitexan (Mesna®) ist im nicht-onkologischen Kontext umstritten. Eine Untersuchung von 1018 Patienten ergab, dass der Einsatz von Uromitexan keinen Einfluss auf das Auftreten einer hämorrhagischen Zystitis hatte [42]. Eine routinemäßige Anwendung als Begleittherapie zu CYC bei immun-mediierten Nierenerkrankungen ist daher nicht obligat. Allerdings sollte die Therapie vormittags verabreicht werden und dabei auf eine ausreichende Flüssigkeitszufuhr bei Ausschluss von Kontraindikationen geachtet werden, um die Verweilzeit der Substanz und ihrer Metabolite in der Blase möglichst kurz zu halten.
3. **Infektionen:** Die Lymphopenie ist eine gefürchtete Komplikation einer höher dosierten Glukokortikoid- sowie CYC-Therapie. Eine kanadische Studie hat hohe Infektionsraten im Rahmen schwerer Lymphopenie-Episoden berichtet [43]. Eine antibiotische Prophylaxe zur Prävention von *Pneumocystis jirovecii* Pneumonie ist bei Patienten mit Systemerkrankungen angezeigt und sollte für die Dauer der Therapie fortgesetzt werden. Trimethoprim-Sulfamethoxazol hat sich als Prophylaxe bewährt und reduziert auch das Auftreten anderer schwerer bakterieller Infektionen [25]. Bezüglich Dosierungsempfehlungen siehe Punkt „Prophylaxe gegen *Pneumocystis jirovecii* Pneumonie“
4. **Malignomrisiko:** Als CYC noch als Induktions- und Erhaltungstherapie sowie zur Behandlung von Rezidiven eingesetzt wurde, kam es häufig zu hohen kumulativen Dosen. Eine Gesamtdosis > 36 g war mit einem Auftreten von weißem Hautkrebs, Harnblasenkrebs sowie von myeloiden Leukämie assoziiert [44]. Durch die deutliche Reduktion der CYC-Exposition besteht nur mehr eine Assoziation mit weißem Hautkrebs, wobei dieses Risiko mit zunehmender Gesamtdosis ansteigt. Eine Interaktion mit einer Azathioprin-Erhaltungstherapie kann in diesen Fällen nicht ausgeschlossen werden [45].

Laut KDIGO sollte die CYC-Gesamtdosis 200 mg/kg Körpergewicht oder 25 g absolut nicht überschreiten (für die Erhaltung der Fertilität wird eine Kumulativdosis von 10 g angegeben). Es ist daher sinnvoll, schon bei Therapiebeginn eine prospektive Erfassung der Kumulativdosis miteinzuplanen [12]. Bezüglich seltenerer Komplikationen und Besonderheiten dieser Therapie sei auf eine umfassende Übersichtsarbeit von Brummaier et al. verwiesen [35].
